# Perception Regarding Knowledge of COVID-19 Prevention in a Sample of a Middle Eastern and North African (MENA) Community in Houston, Texas, USA

**DOI:** 10.3390/ijerph19010524

**Published:** 2022-01-04

**Authors:** Jenna Zamil, Fatin Atrooz, Zahra Majd, Sarah Zeidat, Ghalya Alrousan, Susan Abughosh, Samina Salim

**Affiliations:** 1 College of Natural Sciences and Mathematics, University of Houston, Houston, TX 77204, USA; jennazamil@gmail.com; 2 Department of Pharmacological and Pharmaceutical Science, College of Pharmacy, University of Houston, Houston, TX 77204, USA; fyatrooz@uh.edu (F.A.); sarazeidat@gmail.com (S.Z.); galrousa@central.uh.edu (G.A.); 3 Pharmaceutical Health Outcomes and Policy, College of Pharmacy, University of Houston, Houston, TX 77204, USA; majd.zahra@gmail.com (Z.M.); smabugho@central.uh.edu (S.A.)

**Keywords:** Syrian refugees, stress, trauma, displacement, refugee mental health

## Abstract

(1) Background: Knowledge of COVID-19 prevention among communities is the first step towards protective behaviors. The objective of this study was to assess COVID-19 prevention knowledge among a Middle Eastern and North African community in Houston, Texas. (2) Methods: A cross-sectional study was conducted using a validated quantitative survey; survey questions consisted of three parts: COVID-19 specific questions, general health questions, and sociodemographic questions. A multivariable logistic regression model was used to determine predictors of perception of knowledge on preventing COVID-19 spread. The outcome of interest comprised of “good/excellent” versus “average and below” knowledge. (3) Results: A total of 366 participants (66.39% males) completed the survey. A univariate analysis demonstrated significant differences in self-reported COVID-19 prevention knowledge among those with and without health insurance, different ages, level of knowledge, and perceived severity of COVID-19 infection. In the multivariate logistic regression, two predictors were identified: those in the 18–25-year-old group were more likely to have “excellent/good” knowledge on COVID-19 spread compared to the ≥40-year-old group (OR: 6.36; 95% CI: 1.38, 29.34). Those who somewhat agree with knowing how to protect themselves from COVID-19 were more likely to have “excellent/good” knowledge of preventing COVID-19 spread compared to those that neither agree nor disagree or disagree (OR: 7.74; 95% CI: 2.58, 23.26). (4) Conclusions: Younger adults reported higher knowledge of COVID-19 prevention.

## 1. Introduction

The catastrophic effects of the coronavirus disease 2019 (COVID-19), the highly contagious infectious disease caused by severe acute respiratory syndrome coronavirus 2 (SARS-CoV-2), has resulted in more than 5.0 million deaths worldwide, emerging as the most consequential global health crisis since the influenza pandemic of 1918 (WHO, November 2021 [1]). The COVID-19 disease is characterized by rapid transmission, fostered by close contact with an infected person [2,3]. Despite the unprecedented national measures in containing the outbreak, the success of prevention efforts is largely dependent on public behavior. Public adherence to preventive measures established by the Centers for Disease Control and Prevention (CDC) is critical in curbing the spread of the disease. Public adherence is influenced by knowledge and attitudes towards COVID-19. More than 40 million people living in the United States (US) are foreign-born including the refugee, immigrant, and migrant (RIM) populations who have settled in the US for a variety of different reasons [4,5]. The RIM group, critical for America’s social, cultural, and economic vitality, is a heterogenous mixture of different ethnicities, cultures, and languages, thus representing a broad range of socioeconomic statuses, educational levels, occupations, and English proficiency levels [6], consequently, resulting in considerable variations in awareness, attitudes, and practices toward COVID-19 [7].

In this study, we have focused on immigrants from the Middle East and North Africa (MENA) region residing in Houston, Texas. The MENA community is a historically understudied group in the US, partly due to the lack of a well-defined taxonomic category in the US Census. Individuals of MENA descent are classified as “Caucasian” or “White” in the US Census [8]. Consequently, health behavior research data of the MENA group is mixed with Caucasian data, thus masking MENA-specific public health behaviors. Only a handful of studies have examined MENA health behaviors [9,10,11,12], emphasizing the need to conduct more systematic studies in this important group. The estimated number of MENA individuals in the US is more than 3.5 million [13], and Texas has the fourth largest MENA population in the country, with over 281,000 MENA Texas residents [14,15]. Houston reportedly has 98,300 MENA residents (Houston Chronicle [16]). According to the Arab American Institute, the largest population of MENA Texans reside in Harris and Fort Bend counties, including the Katy and Sugar Land area (The Texas Tribune, 2020 [17]). The present COVID-19 survey, therefore, targeted MENA individuals residing in the Harris and Fort Bend Counties in the Houston Metro area.

To encourage protective measures, knowledge on COVID-19 is critical. Several studies have pointed out a close relationship between sociodemographic characteristics and COVID-19 knowledge and vaccination status [18]. Among Middle Easterners, prior perceptions, location, and education level showed a possible connection with COVID-19 knowledge. For example, among medical students in Iran, COVID-19 knowledge was found to be accurate, with 79.6% of participants demonstrating high COVID-19 knowledge and over 90% demonstrating high levels of protective behavior [19]. However, a study evaluating COVID-19 knowledge among MENA residents in Dearborn, Michigan, found that the United States death toll of COVID-19 was “severely underestimated” by study participants, who predicted over 400,000 fewer deaths nationally than currently recorded [12]. These results suggest a need to evaluate existing knowledge and develop interventions to increase COVID-19 knowledge among Middle Eastern Americans. In Houston, the subpopulations of MENA immigrants with the lowest COVID-19 prevention knowledge levels have not yet been identified, which informs our research. Understanding perceptions and knowledge regarding COVID-19 and how to protect against infection among Middle Eastern Americans is the first step to designing effective interventions to promote vaccination as well as other behaviors, such as distancing, that can curb the virus in the community. Therefore, the objective of this study was to evaluate perceptions of knowledge on how to protect against COVID-19 among the MENA group and to identify subpopulations within this immigrant community who would benefit the most from educational outreach programs and vaccine promotion measures.

## 2. Materials and Methods

This is a cross-sectional survey-based study conducted between July and August 2021 among MENA members residing in the Houston area. All methods used and survey questionnaires utilized were approved by the Institutional Review Board (IRB, #STUDY00003078) Committee for the Protection of Human Subjects, University of Houston, Houston, TX, USA.

***Study participants*:** The present study was conducted during the COVID-19 pandemic; therefore, all institutional guidance and restrictions were followed; all communications were over the phone, text messaging, or online platforms (Zoom, TEAMS). After our study protocol was approved by the UH-IRB Committee, an anonymous, digital bilingual (English and Arabic) survey was created using a generalized survey service known as RedCap, and the survey link was sent to the MENA community through Houston-based 501 (C) (3) non-profit organization MultiCultural Center (MCC), Webster, TX (http://www.multiculturalcenter.net/, accessed on 27 July 2021). Date of survey dissemination 07/27/2021. MCC works with the MENA community in the Houston area. The RedCap survey dissemination was facilitated by MCC coordinator Dr. Uzma Khan (PharmD) by sharing the survey link to the MCC listserv. A study fellow, students, and research assistant also recruited participants using their social media pages. Anyone who was willing to participate in the survey and identified themselves as a Houston resident and belonged to the MENA community was included in the survey. Participants were included in the study by stating their country of origin from the Middle East and North Africa. The survey was distributed through a community organization listserv MCC, which serves the MENA community.

***Inclusion Criteria*:** MENA adult men and women (country of origin: Algeria, Bahrain, Egypt, Iran, Iraq, Israel–Palestine, Jordan, Kuwait, Lebanon, Libya, Morocco, Oman, Qatar, Saudi Arabia, Syria, Tunisia, United Arab Emirates, and Yemen), 18 years old and above, who are settled in the Houston area were included in this study. *Exclusion criteria*: MENA individuals under the age of 18. MENA individuals who are residing out of the Houston area and non-members of the MENA group.

***Survey*:** The survey was created by Dr. Nadia Abuelezam [11,20], William F. Connell School of Nursing, Boston College, MA. The survey comprised of previously validated questions that were originally written in English and translated into Arabic by a bilingual Arabic translator. The first page of the survey included the consent form, which has comprehensive information about the study and the principal investigator’s contact information. In the consent form, participants were asked to provide consent for participation in the study. The quantitative survey questions consisted of three parts: COVID-19 specific questions, general health questions, and sociodemographic questions. COVID-19-related factors included: prior COVID-19 infection, perceived infection risk, perceived infection severity, perceived knowledge regarding self-protection from the COVID-19 infection, and perceived ability to avoid the infection. Health questions included: health insurance status, overall health status, smoking status, alcohol consumption, and disease comorbidities such as hypertension, hypercholesterolemia, obesity, diabetes, anxiety, and depression. The sociodemographic questions included: age, gender, race/ethnicity, country of origin, religion, immigration status, marital status, educational level, living status, and annual household income. 

***Statistical analysis*:** A descriptive analysis was performed to summarize participants’ sociodemographic, general health, as well as responses to COVID-19-related questions. The frequencies for all categorical variables were calculated and described as percentages. Continuous variables were reported in mean ± standard deviation (SD). Group differences were assessed using chi-square tests. A multivariable logistic regression model was conducted to determine predictors of perception of knowledge on preventing COVID-19 spread. The outcome of interest was having “good/excellent” versus “average and below” knowledge. Covariates included in the model were gender, age, education, marital status, living status, annual house income, religion, smoking status, alcohol consumption, health insurance, overall health perception, and comorbidities such as hypertension, hypercholesterolemia, obesity, and anxiety. COVID-19-related factors included prior infection, perceived infection risk, perceived infection severity, perceived knowledge of self-protection from the COVID-19 infection, and perceived ability to avoid infection. All statistical analyses were performed using SAS version 9.4 (SAS Institute, Cary, NC, USA) at an a priori significance level of 0.05.

Sample size calculation: The G-power 3.1 statistical software was used for sample size estimation [21]. The estimated number of the MENA population in the Houston area is 98,300. Accordingly, a total of 242 subjects were needed to provide 80% power to detect significance with a 0.15 effect size for a chi-square analysis at a 0.05 α-level. Additionally, we calculated that a minimum sample of 308 patients would provide 80% power to detect a 1.5 odds ratio for a two-tailed analysis at α < 0.05 using a logistic regression.

## 3. Results

### 3.1. Demographic Characteristics

Table 1 shows the demographic characteristics and social circumstances of the 366 survey respondents including 243 males (66.39%) and 123 females (33.61%). A majority of the respondents (67.76%) were within the 26–39-year age group. Around one third (27.60%) of the participants reported having some college education and 48.09% reported attainment of college degrees. Of the total respondents, 67.21% were married, 21.58% were single, and 11.20% were widowed or divorced. The average annual income for almost half of the respondents (46.72%) was between $45,000–$100,000. More than half (53.01%) of the respondents identified themselves as Muslims, 34.43% as Christians, and 12.57% as Jewish. Of all the participants, 144 reported smoking cigarettes or hookah (39.34%), and 115 (31.42%) reported drinking alcoholic beverages. Concerning respondents’ overall health, the majority (65.30%) stated as being in very good/good health. Some of the respondents reported the following health issues: hypertension (54; 15.17%), high cholesterol (48; 13.56%), and obesity (52; 14.94%). In total, 29.55% of the respondents, 104 individuals, reported having anxiety.

### 3.2. COVID-19-Related Questions

In this sample, 69 individuals (18.96%) were infected with COVID-19. In parallel, 64 individuals (17.78%) stated that the infection with COVID-19 was extremely severe. Only 41 individuals (11.23%) declared a high likelihood of acquiring COVID-19 infection, and 14.09% reported that avoiding COVID-19 infection was extremely/somewhat difficult, 55.25% of respondents expressed avoiding the infection was extremely/somewhat easy, while 33.61% reported having good knowledge of protection from COVID-19 infection.

Among all variables tested, the univariate analysis showed significant group differences between age groups in knowledge of how to prevent oneself from COVID-19 infection. Additionally, those with and without health insurance, level of knowledge about how to protect oneself against COVID-19, perceived difficulty of avoiding COVID-19 infection, and perceived COVID-19 infection severity also showed a significant difference in knowledge of COVID-19 infection prevention (Table 1).

### 3.3. Multivariable Logistic Regression Analysis

In the multivariate logistic regression analysis, two predictors were identified: those in the age group 18–25 years old were more likely to have “excellent/good” knowledge on preventing COVID-19 spread compared to the age group of 40 years and above (OR: 6.36; 95% CI: 1.38, 29.34). Those who somewhat agree with knowing how to protect themselves from COVID-19 were more likely to have “excellent/good” knowledge of preventing COVID-19 spread compared to those who neither agree nor disagree or disagree (OR: 7.74; 95% CI: 2.58, 23.26) (Table 2).

## 4. Discussion

The present study assessed knowledge, perceptions, and practices related to COVID-19 among Houston MENA residents. In this study, younger participants (18–25 years old) demonstrated more knowledge about COVID-19 transmission and prevention than the older age group, an observation which could be explained by the increased use of social media outlets by this group when compared to the older age group. These results are consistent with several previous studies conducted on COVID-19 knowledge, awareness, and attitudes in the MENA group residing outside of the US [22,23]. According to our study, the 18–25-year-old age group was more likely to report “good/excellent” COVID-19 prevention knowledge than other groups. Interestingly, this age group represents the largest age demographic in the study that is represented on the social media site TikTok, as nearly two-thirds of the site’s user base is under the age of 29. As stated by Ostrovsky et al., the hashtag “coronavirus” generated 93.1 billion views, demonstrating the wide social media coverage of the pandemic [24]. Thus, coronavirus information access and its rapid assimilation through social media could lead to younger adults perceiving a higher knowledge level of COVID-19 prevention than older adult age groups.

Furthermore, young adults are believed to more likely respond to peer influence than older adults [25]. Thus, it is possible that knowledge of preventing COVID-19 among young adults is popularized due to peer influence, providing another factor that could inform our study’s results. However, this rapid assimilation of information among young people also presents the problem of misinformation and raises the possibility of misrepresented information being distributed, allowing a higher perceived level of COVID-19 knowledge than truly gained. This informs the study’s limitations on providing only a self-reported perception of COVID-19 prevention knowledge, as the accuracy of perceived knowledge on preventing COVID-19 was not evaluated. Future studies should focus on the source of young adults’ COVID-19 information and evaluate actual knowledge of COVID-19 in comparison with our results. Additionally, the present study illustrates the need for designing a COVID-19 educational outreach strategy catered towards older adults in the MENA immigrant population, as this group reported lower perceived confidence in their knowledge about preventing COVID-19. Clearly, the healthcare needs of the elderly MENA community in general, and COVID-19 prevention strategies in particular, need attention. Surprisingly, only a handful of studies have examined the healthcare needs of older MENA Americans [9,26,27]. Thus, more work is needed in this area to understand how to best care for aging MENA Americans and to ensure effective and feasible COVID-19 prevention strategies for this group.

Findings from our study also suggest that individuals who reported relatively adequate knowledge on how to protect themselves from COVID-19 infection also reported higher levels of COVID-19 prevention knowledge. In a study by Riad Abanoub et al., knowledge about COVID-19 showed a positive correlation with protective behaviors against COVID-19 [28]. These results underscore the importance of adopting language-tailored culturally competent COVID-19 educational programs for MENA immigrants, as customized programs may lead to greater acceptance of COVID-19 vaccinations, higher knowledge, and improved practice of protective health behaviors. Such language-tailored interventions by incorporating cultural beliefs may also mitigate apprehensions about COVID-19. Constant monitoring of vaccination status, COVID-19 prevalence, the latest scientific evidence, and public opinion monitoring are needed for effective disease prevention [18].

## 5. Conclusions

Our results can help inform the direction of future educational and public health endeavors in Houston’s MENA community towards populations in need, allowing MENA adults to increase their knowledge on protection against COVID-19 and thus, allow for apprehensions about vaccination and other protective measures to curb the spread of this virus within and beyond the MENA community. Finally, more attention is needed to better understand the social and health needs of the MENA group in the context of the existing racial and minority health landscape in the US. This is critical for better understanding of immigrant health in the US.

## 6. Limitations

Several limitations of this study can be reported. The cross-sectional design precludes cause-and-effect relationships. Findings of this study are based on self-reported data in a relatively small sample size and should be interpreted given these limitations. The MENA community is a heterogenous group including individuals of Arab, Turkish, and Persian descent, with varying socioeconomic status. However, our sample included predominantly Arab members; therefore, COVID-19 vaccination-related behaviors among the Turkish and Persian community were not discussed. Future studies are recommended to evaluate the knowledge of COVID-19 prevention among the MENA community using different study designs and larger samples. Finally, given the scarce literature in this population, this cross-sectional study served as a hypothesis-generating study using a validated survey of demographics and health-related and knowledge-based variables, but it was not built on a fully developed conceptual framework. Taking our findings into consideration, more conceptually based future work is warranted and is currently in progress.

## Figures and Tables

**Table 1:** Baseline demographic and characteristics of knowledge on preventing COVID-19 spread.

	Total (%)	Knowledge—Prevent COVID-19 Spread		*p* Value
		Excellent/ Good	Average/ Poor/ Terrible	
**Variable**				
**Gender**				
Males	243 (66.39)	182 (65.47)	61 (69.32)	0.5051
Females	123 (33.61)	96 (34.53)	27 (30.68)	
**Age (years)**				
18–25	85 (23.22)	72 (25.90)	13 (14.77)	0.0377 *
26–39	248 (67.76)	185 (66.55)	63 (71.59)	
≥40	33 (9.02)	21 (7.55)	12 (13.64)	
**Education**				
High school or less	50 (13.59)	37 (13.31)	13 (14.77)	0.6250
Some college or associate degree	101(27.45)	74 (26.62)	27 (30.68)	
College degree	176 (48.09)	139 (50.00)	37 (42.05)	
Graduate degree	39 (10.66)	28 (10.07)	11 (12.50)	
**Marital Status**				
Married	246 (67.21)	186 (66.91)	60 (68.18)	0.1329
Single	79 (21.59)	65 (23.38)	14 (15.91)	
Divorced/Separated	41 (11.20)	27 (9.71)	14 (15.91)	
**Living Status**				
Own	251(68.58)	198 (71.22)	53 (60.23)	0.0528
Rent	115 (31.42)	80 (28.78)	35 (39.77)	
**Annual Household Income**				
<$25,000	59 (16.12)	43 (15.47)	16 (18.18)	0.2790
$25,000 to <$45,000	94 (25.68)	73 (26.26)	21 (23.86)	
$45,000 to <$65,000	97 (26.50)	69 (24.26)	28 (31.82)	
$65,000 to <$100,000	74 (20.22)	56 (20.14)	18 (20.45)	
≥$100,000	42 (11.48)	37 (13.31)	5 (5.68)	
**Religion**				
Christian	126 (34.43)	102 (36.69)	24 (27.27)	0.2676
Muslim	194 (53.01)	142 (51.08)	52 (59.09)	
Jewish/other	46 (12.57)	34 (12.23)	12 (13.64)	
**Smoke**				
No	222 (60.66)	161 (57.91)	61 (69.32)	0.0563
Yes	144 (39.34)	117 (42.09)	27 (30.68)	
**Drink Alcohol**				
No	251 (68.58)	189 (67.99)	62 (70.45)	0.6637
Yes	115 (31.42)	89 (32.01)	26 (29.55)	
**Health Insurance**				
Yes	291 (79.51)	228 (82.01)	63 (71.59)	0.0348 *
No	75 (20.49)	50 (17.99)	25 (28.41)	
**Overall Health**				
Excellent	105 (28.69)	83 (29.86)	22 (25.00)	0.3036
Very Good/Good	239 (65.30)	181 (65.11)	58 965.91)	
Fair/Poor	22 (6.01)	14 (5.04)	8 99.09)	
**Hypertension**				
Yes	54 (15.17)	45 (16.54)	9 (10.71)	0.1929
No	302 (84.83)	227 (83.56)	75 (89.29)	
**High Cholesterol**				
Yes	48 (13.56)	32 (11.94)	16 (18.60)	0.1163
No	306 (86.44)	236 (88.06)	70 (81.40)	
**Obesity**				
Yes	52 (14.94)	41 (15.36)	11 (13.58)	0.6946
No	296 (85.06)	226 (84.64)	70 (86.42)	
**Anxiety**				
Yes	104 (29.55)	82 (30.26)	22 (27.16)	0.5918
No	248 (70.45)	189 (69.74)	59 (72.84)	
**Infected with COVID-19**				
Yes	69 (18.96)	52 (18.71)	17 (19.77)	0.8261
No	295 (81.04)	226 (81.29)	69 (80.23)	
**COVID-19 Infection Risk**				
Extremely Likely	41 (11.23)	31 (11.19)	10 (11.36)	0.7487
Somewhat Likely	145 (39.73)	113 (40.79)	32 (36.36)	
Neither Likely nor Unlikely/ Somewhat or Extremely Unlikely	179 (49.04)	133 (48.01)	46 (52.27)	
**COVID-19 Infection Severity**				
Extremely Severe	64 (17.78)	55 (2015)	9 (10.34)	0.0200 *
Somewhat Severe	156 (43.33)	122 (44.69)	34 (39.08)	
Neither Severe nor Mild	95 (26.39)	62 (22.71)	33 (37.93)	
Somewhat Mild/Extremely Mild	45 (12.50)	34 (12.49)	11 (12.64)	
**Know Self-Protection from COVID-19 Infection**				
Strongly Agree	123 (33.61)	107 (38.49)	16 (18.18)	0.0001 **
Somewhat Agree	169 (46.170	138 (49.64)	31 (35.23)	
Neither Agree nor Disagree/ Somewhat/Strongly Disagree	74 (20.22)	33 (11.87)	41 (46.59)	
**Avoiding COVID-19 Infection**				
Extremely/Somewhat Easy	200 (55.250	164 (59.42)	36 (41.86)	0.0055 *
Neither Easy nor Difficult	111 (30.66	73 (26.45)	38 (44.19)	
Somewhat/Extremely Difficult	51 (14.09)	39 (14.13)	12 (13.95)	

* Significant *p* value from chi square < 0.05; ** Significant *p* value from chi square < 0.001.

**Table 2:** Multivariable logistic regression of knowledge on preventing COVID-19 spread.

Excellent/Good Knowledge vs. Average/Poor/Terrible		
	OR (95% CI)	*p* Value
**Variable**		
**Gender**		
Male vs. Female	0.75 (0.37–1.54)	0.4341
**Age**		
18–25 vs. ≥40	6.36 (1.38–29.34)	0.0223 *
26–39 vs. ≥40	2.99 (0.86–10.40)	0.6771
**Education**		
≤ High School vs. Graduate Degree	1.89 (0.40–8.94)	0.745
Some College vs. Graduate Degree	1.44 (0.41–5.13)	0.6813
College Degree vs. Graduate Degree	2.63 (0.81–8.53)	0.1008
**Marital Status**		
Married vs. Divorced/Separated	2.60 (0.92–7.39)	0.1252
Single vs. Divorced/Separated	1.94 (0.60–6.32)	0.6962
**Living Status**		
Own vs. Rent	0.95 (0.44–2.08)	0.9054
**Annual Household Income**		
<$25,000 vs. ≥$100,000	0.08 (0.01–0.47)	0.1091
$25,000 to <$45,000 vs. ≥$100,000	0.12 (0.02–0.64)	0.4358
$45,000 to <$65,000 vs. ≥$100,000	0.12 (0.02–0.57)	0.3838
$65,000 to <$100,000 vs. ≥$100,000	0.08 (0.02–0.44)	0.0985
**Religion**		
Christian vs. Jewish/Other	2.27 (0.76–6.76)	0.0978
Muslim vs. Jewish/Other	1.30 (0.47–3.60)	0.6994
**Smoke**		
No vs. Yes	0.48 (0.23–1.02)	0.0573
**Drink Alcohol**		
No vs. Yes	1.43 (0.66–3.11)	0.3647
**Health Insurance**		
Yes vs. No	0.84 (0.36–1.93)	0.6775
**Overall Health**		
Excellent vs. Fair/Poor	1.78 (0.40–7.94)	0.9372
Very Good/Good vs. Fair/Poor	2.94 (0.74–11.73)	0.0753
**Hypertension**		
Yes vs. No	3.06 (0.92–10.23)	0.0692
**High Cholesterol**		
Yes vs. No	0.83 (0.26–2.71)	0.7615
**Obesity**		
Yes vs. No	1.77 (0.62–5.08)	0.29
**Anxiety**		
Yes vs. No	1.17 (0.53–2.59)	0.6904
**Infected with COVID-19**		
Yes vs. No	0.54 (0.23–1.31)	0.1725
**COVID-19 Infection Risk**		
Extremely Likely vs. Neither Likely nor Unlikely/Somewhat Unlikely	1.99 (0.48–8.34)	0.2509
Somewhat Likely vs. Neither Likely nor Unlikely/Somewhat Unlikely	0.78 (0.38–1.63)	0.1908
**COVID-19 Infection Severity**		
Extremely Severe vs. Somewhat/Extremely Mild	1.88 (0.47–7.45)	0.2369
Somewhat Severe vs. Somewhat/Extremely Mild	0.92 (0.31–2.74)	0.454
Neither Severe nor Mild vs. Somewhat/Extremely Mild	0.98 (0.32–2.99)	0.6432
**Know Self-Protection from COVID-19 Infection**		
Strongly Agree vs. Neither Agree nor Disagree/	7.74 (2.58–23.26)	0.0043 *
Somewhat Disagree/Strongly Disagree		
Somewhat Agree vs. Neither Agree nor Disagree/	4.49 (1.84–10.96)	0.177
Somewhat Disagree/Strongly Disagree		
**Avoiding COVID-19 Infection**		
Extremely Easy/Somewhat Easy vs. Somewhat Difficult/Extremely Difficult	1.02 (0.39–2.98)	0.3377
Neither Easy nor Difficult vs. Somewhat Difficult/Extremely Difficult	0.49 (0.17–1.42)	0.0629

* Significant *p* value from chi square < 0.05.

## Data Availability

The data presented in this study are available on request from the corresponding author.

## Supplementary Materials

The following supporting information can be downloaded at: https://www.mdpi.com/article/10.3390/ijerph19010524/s1. Survey Questionnaire.
