# Supplementary material for: Perception Regarding Knowledge of COVID-19 Prevention in a Sample of a Middle Eastern and North African (MENA) Community in Houston, Texas, USA
Source: Int J Environ Res Public Health. 2022 Jan 4;19(1):524. doi: 10.3390/ijerph19010524 (PMC8744562; doi:10.3390/ijerph19010524)
Supplement: Supplementary file 1 [file ijerph-19-00524-s001.zip › ijerph-1493576-supplementary.pdf]

Project Home | Project Setup | Online Designer | Data Dictionary | Codebook

Online Designer

Since this project is currently in PRODUCTION, changes will not be made in real time. Tell me more

Submit Changes for Review

Fields to be added: **0** / Total resulting field count: **129**
Fields to be deleted: **0** / Existing field count: **129**

Remove all drafted changes

View detailed summary of all drafted changes

Create snapshot of instruments

Last snapshot: never ?

VIDEO: How to use this page

This page allows you to build and customize your data collection instruments one field at a time. You may add new fields or edit existing ones. New fields may be added by clicking the Add Field buttons. You can begin editing an existing field by clicking on the **Edit** icon. If you decide that you do not want to keep a field, you can simply delete it by clicking on the **Delete** icon. To reorder the fields, simply **drag and drop** a field to a different position within the form below.

Return to list of instruments | Survey settings | « Previous instrument | Next instrument »

Current instrument: **Consent form**

Preview instrument

Add Field | Add Matrix of Fields | Import from Field Bank

Variable: cover_letter_e

Title of research study: Assessing awareness, access, and acceptance of COVID-19 vaccinations in the Middle Eastern and North African (MENA) community of the Greater Houston area.

Investigator: Name: Samina Salim, PhD
Department: Pharmacological and Pharmaceutical Sciences
Telephone Number: 713-743-1776
Email Address: ssalim2@central.uh.edu

Key Information:
The following focused information is being presented to assist you in understanding the key elements of this study, as well as the basic reasons why you may or may not wish to consider taking part. This section is only a summary; more detailed information, including how to contact the research team for additional information or questions, follows within the remainder of this document under the "Detailed Information" heading.

What should I know about a research study?
• Someone will explain this research study to you.
• Taking part in the research is voluntary; whether or not you take part is up to you.
• You can choose not to take part.
• You can agree to take part and later change your mind.
• Your decision will not be held against you.
• You can ask all the questions you want before you decide, and can ask questions at any time during the study.

We invite you to take part in a research study, the purpose of which is, to contribute to a greater understanding of the health needs of Arab Americans during the COVID-19 outbreak. You have been asked to participate in this study because you meet the following criteria:

- You are an adult (≥ 18 years)
- You are of Arab origin living in the United States in the greater Houston area

Your participation in the research involves completing a survey which will include questions about demographic information, general health, and the coronavirus (COVID-19). The survey should take approximately 10-15 minutes to complete and will be administered online. We hope to gain a better understanding of how Arab Americans are affected by COVID-19. As a token of appreciation for completing the survey, we will provide a $10 Amazon e-gift card for your participation if you are interested in receiving one.

Detailed Information:

The following is more detailed information about this study, in addition to the information listed above.

Why is this research being done?

Little is known about the health risks of Arab Americans with regard to the COVID-19 pandemic. Arab Americans may have a number of risk factors that put them at increased risk of exposure to and complications from COVID-19. There has never been a survey done to assess Arab American health needs in the United States of America perhaps due to a lack of funding and/or an inability to capture Arab Americans from standard race and ethnicity survey measures. Additionally, because Arab Americans are classified as White on racial and ethnic surveys, there is no way to extract information on Arab American health from existing surveys on COVID-19. We therefore are conducting one of the first representative surveys on Arab American health in the Houston area, to understand their health needs and risks, specifically related to COVID-19.

The study is funded through the 2021 Houston Global Health Collaborative Student Research and Service Grant awarded to college of pharmacy P4 student Sarah Zeidat, under the mentorship of Dr. Samina Salim (PI). The purpose is to examine the understanding, access and uptake of the COVID-19 vaccination among Arab Americans (MENA group in the Houston area). The participants will be asked to complete a survey which will include questions about coronavirus (COVID-19), general health questions, and demographic information. The survey should take approximately 10-15 minutes to complete and will be administered on-line. Through the survey, we will examine COVID-19 vaccine hesitancy, awareness and acceptance in the MENA group settled in the greater Houston area.

How long will the research last?

Your participation ends as soon as you finish the survey. The participation is 100% virtual (online).

How many people will be studied?

We expect to enroll about 250 people in this research study.

What happens if I say yes, I want to be in this research?

If you agree to participate in this study, you will participate one time, in an online survey questionnaire in your preferred language, English or Arabic.

What happens if I do not want to be in this research?

You can choose not to take part in the research and it will not be held against you. If you are a student, a decision to take part or not, or to withdraw from the research will have no effect on your grades or standing with the University of Houston.

What happens if I say yes, but I change my mind later?

You can leave the research at any time and it will not be held against you. If you decide to leave the research, contact the investigator so that the investigator can update the record in the research study. If you stop being in the research, already collected data that still includes your survey information may not be removed from the study record.

Is there any way being in this study could be bad for me?

There are no foreseeable risks related to the procedures conducted as part of this study. We do not expect any risks related to the research activities. If you choose to take part in the survey and undergo a negative event you feel is related to the study, please contact the researcher/study team PI Dr. Samina Salim. However, loss of confidentiality regarding research information is a possibility, although the risk is small. Efforts will be made to limit the use and disclosure of your personal information, to organizations that might need to review this information.

Will I get anything for being in this study?

A $10 amazon gift card will be provided for participation in the survey questionnaire to interested individuals.

Will being in this study help me in any way?

There are no known benefits to you from your taking part in this research. However, possible benefits to others include advancing knowledge and information about Arab health in the US.

What happens to the information collected for the research?

Your taking part in this project is anonymous, and information you provide cannot be linked to your identity. We may publish the results of this research.

Can I be removed from the research without my OK?

The person in charge of the research study or the sponsor can remove you from the research study without your approval if your survey was incomplete.

Who can I talk to?

If you have questions, concerns, or complaints, or think the research has hurt you, you should talk to the research team at

PRINCIPAL INVESTIGATOR:

Name: Samina Salim

Department: Pharmacological and Pharmaceutical Sciences

Telephone Number: 713-743-1776;

Email Address: ssalim2@central.uh.edu

This research has been reviewed and approved by the University of Houston Institutional Review Board (IRB). You may also talk to them at (713) 743-9204 or cphs@central.uh.edu if:

- Your questions, concerns, or complaints are not being answered by the research team.
- You cannot reach the research team.
- You want to talk to someone besides the research team.
- You have questions about your rights as a research subject.
- You want to get information or provide input about this research.

Add Field | Add Matrix of Fields | Import from Field Bank

Variable: agree_e

I have read the consent information and agree to take part in the research

* must provide value

- ○ Yes
- ○ No

Add Field | Add Matrix of Fields | Import from Field Bank

Project Home | Project Setup | Online Designer | Data Dictionary | Codebook

Online Designer

Since this project is currently in PRODUCTION, changes will not be made in real time. Tell me more

Submit Changes for Review

Fields to be added: **0** / Total resulting field count: **129**
Fields to be deleted: **0** / Existing field count: **129**

Remove all drafted changes

View detailed summary of all drafted changes

Create snapshot of instruments

VIDEO: How to use this page

Last snapshot: never [?]

This page allows you to build and customize your data collection instruments one field at a time. You may add new fields or edit existing ones. New fields may be added by clicking the Add Field buttons. You can begin editing an existing field by clicking on the **Edit** icon. If you decide that you do not want to keep a field, you can simply delete it by clicking on the **Delete** icon. To reorder the fields, simply **drag and drop** a field to a different position within the form below.

Return to list of instruments | Survey settings | « Previous instrument | Next instrument »

Current instrument: **Consent form (Arabic)**

Return to edit view

NOTE: Please be aware that branching logic and calculated fields will not function on this page. They only work on the survey pages and data entry forms.

عنوان الدراسة البحثية: تقييم الوعي وإمكانية الوصول وقبول مطعوم COVID-19 في مجتمع الشرق الأوسط وشمال إفريقيا (MENA) في منطقة هيوستن الكبرى.

الباحث الأساسي:

الاسم: الدكتورة سامينا سالم

القسم : الصيدلة وعلم الأدوية

رقم الهاتف: 1776-743-713

البريد الالكتروني: ssalim2@central.uh.edu

معلومات أساسية:

نقدم لكم المعلومات التاليه لمساعدتكم على فهم العناصر الأساسيه في هذه الدراسة, كذلك لتزويدكم بالأسباب الرئيسية التي قد تجعلكم ترغبون أو لا ترغبون بأن تكونوا جزءا من هذه الدراسة. هذا الفصل فقط ملخص , ستجدون معلومات أوفى عن هذه الدراسة و كيفية الاتصال بفريق البحث اذا كان لديكم أي استفسار او كنتم ترغبون بالحصول على معلومات اضافية لاحقاً في فصل "معلومات تفصيلية".

ماذا علي ان أعرف عن هذه الدراسة البحثية ؟

- سيقوم احدهم بتوضيح هذه الدراسة البحثية لك.
- المشاركة في البحث تطوعية, فالامر يعود لك بالايجاب اوالرفض.
- يمكك اختيار عدم المشاركة .
- يمكنك الموافقة على المشاركة ولك الحق في تغيير رأيك بأي وقت لاحقا.
- لن يكون لقرارك اي اثر عليك.
- يحق لك طرح اي سؤال قبل قرار المشاركة بالدراسة او حتى لاحقا في أي وقت اثناء الدراسة.

أنت مدعو للمشاركة في بحث يهدف للحصول على فهم أفضل للاحتياجات الصحية للأمريكيين العرب أثناء تفشي COVID-19 .يُطلب منك المشاركة في الدراسة لأنك تنطبق عليك المواصفات التالية:

- شخص بالغ (العمر 18+ سنة),

-من أصل / ثقافة / عرق من بلد يتحدث العربية و تعيش في الولايات المتحدة في منطقة هيوستن.

بشكل عام ، تتضمن مشاركتك في البحث إكمال استبيان يتضمن أسئلة حول فيروس كورونا (COVID-19) وأسئلة صحية عامة ومعلومات ديموغرافية. نتوقع أن يستغرق ملء الاستبيان حوالي 15-10 دقيقة, وسيتم إدارته عبر الإنترنت.

نأمل في الحصول على فهم أفضل لكيفية تأثر الأمريكيين العرب بـ COVID-19 . كتقدير لاستكمال الاستبيان ، سنقدم بطاقة هدايا بقيمة 10 دولارات أمريكية مقابل مشاركتك, اذا كنت مهتما بالحصول على واحدة.

معلومات تفصيلية:

فيما يلي معلومات أكثر تفصيلاً حول هذه الدراسة ، بالإضافة إلى المعلومات المذكورة أعلاه

لماذا يتم عمل هذه الدراسة؟

لا يُعرف سوى القليل عن المخاطر الصحية للأمريكيين العرب فيما يتعلق بوباء كوفيد 19

قد يكون لدى الأمريكيين العرب عدد من العوامل التي تعرضهم لخطر متزايد للتعرض لمضاعفات كوفيد 19 . لم يتم إجراء أي دراسة استقصائية لتقييم الاحتياجات الصحية للأمريكيين العرب في الولايات المتحدة الأمريكية ربما بسبب نقص التمويل و / أو عدم القدرة على التقاط العرب الأمريكيين من مقاييس استطلاعات العرق والنسب القياسي. بالإضافة إلى ذلك ، نظرًا لتصنيف الأمريكيين العرب على أنهم أبيض في الدراسات الاستقصائية العرقية والإثنية ، فلا توجد طريقة لاستخراج معلومات عن صحة الأمريكيين العرب من الاستطلاعات الحالية. لذلك نجري واحدة من أولى الدراسات الاستقصائية التمثيلية حول صحة الأمريكيين العرب في منطقة هيوستن ، لفهم احتياجاتهم الصحية ومخاطرهم ، وخاصة المتعلقة بكوفيد 19. الغرض من هذه الدراسة (الممولة

من خلال منحة هيوستن العالمية للبحوث والخدمات التعاونية للصحة العالمية ، 2021 الممنوحة لطالبة كلية الصيدلة سارة زيدات ، و المشرفة و الباحث الرئيسي: الدكتورة سامينا سالم) هو فحص الفهم والوصول واخذ التطعيم ضد فيروس كورونا بين الأمريكيين العرب (مجموعة الشرق الأوسط وشمال إفريقيا تشمل اللاجئين في منطقة هيوستن). سيُطلب من المشاركين إكمال استبيان يتضمن أسئلة حول فيروس كورونا (كوفيد 19) وأسئلة صحية عامة ومعلومات ديموغرافية. يستغرق اللاستبيان حوالي 10-15 دقيقة لإكماله وسيتم إدارته عبر الإنترنت. من خلال الاستطلاع ، سنفحص تردد أخذ المطعوم والوعي والقبول في مجموعة الشرق الأوسط وشمال إفريقيا المستقرة في منطقة هيوستن الكبرى

كم من الوقت يحتاج هذا البحث

تنتهي مشاركتك بمجرد الانتهاء من الاستبيان المشاركة. المشاركة عبر الانترنت 100٪ (أونلاين).

كم شخص سيشارك بالدراسه؟

نتوقع تسجيل حوالي 250 شخصًا في هذه الدراسة البحثية.

ماذا يحدث إذا قلت نعم ، أريد أن أكون في هذا البحث؟

إذا وافقت على المشاركة في هذه الدراسة ، فسوف تشارك في استبيان استطلاع عبر الإنترنت بلغتك المفضلة ، الإنجليزية أو العربية.

ماذا يحدث إذا كنت لا ارغب المشاركة في هذا البحث ؟

يمكنك اختيار عدم الموافقة على المشاركة في هذا البحث, ولن يتم اعتبار ذلك ضدك. ان اختيار عدم المشاركة لن يترتب عليه اي عقوبة أو خسارة في الفائدة المستحقة لك من أي جهة. اذا كنت طالباً في جامعة هيوستن , قرارك بالموافقة على المشاركة أو عدم الموافقة , أو الانسحاب بعد الموافقة , لن يؤثر على علاماتك او موقعك بالجامعة . الاختيار البديل للمشاركة بالنسبة لك هو عدم المشاركة.

ماذا سيحدث لو قلت نعم لكنني غيرت رأيي لاحقا ؟

يمكنك سحب موافقتك على المشاركة في الدراسة والإنسحاب من البحث في اي وقت, و لن يحسب هذا ضدك. ان كان خيارك التوقف عن المشاركة في البحث في , تواصل مع فريق البحث لتعديل بيانات الدراسة.

أن كان خيارك التوقف عن المشاركة في البحث، سيتم حذف جميع البيانات المتعلقة بكم, والتي تتضمن اسمك و جميع المعلومات الشخصية, من سجلات الدراسة.

هل من الممكن أن ينتج عن المشاركة بهذه الدراسة شيء سيء بالنسبة لي؟

لا نتوقع حدوث أي مخاطر متعلقة بالمشاركة بالبحث. إذا كان خيارك المشاركة بالبحث ثم واجهت أحداث سلبية تعتقد انها قد تكون متعلقة بالمشاركة بالبحث, يرجى الاتصال بفريق البحث المذكور و الباحث الاساسي الدكتورة سمينه سالم. ومع ذلك, هناك احتمال فقدان بعض الخصوصية المتعلقة بالمعلومات المحصَّلة من البحث, بالرغم من أن هذا الاحتمال ضئيل جداً, سنبذل أقصى ما بوسعنا للحد من افشاء المعلومات الشخصيّة, بما يتضمن نتائج البحث, للمنظمات التي قد تحتاج مراجعة هذه المعلومات.

هل سأحصل على اي شيء لقاء المشاركة في هذه الدراسة ؟

سيتم تقديم بطاقة هدايا بقيمة 10 دولارًا للمشاركة بتعبئة الاستبيانات, للمهتمين بالحصول على واحدة.

هل لمشاركتي في هذه الدراسة فائدة بأي شكل من الاشكال ؟

لا يمكننا أن نعد بأي فوائد لك أو للآخرين من مشاركتك في هذا البحث. ومع ذلك ، تشمل الفوائد المحتملة اكتساب المعرفة والمعلومات حول صحة العرب في الولايات المتحدة.

ماذا يحدث للمعلومات التي تم جمعها للبحث ؟

مشاركتك في هذا المشروع مجهولة المصدر ، والمعلومات التي تقدمها لا يمكن ربطها بهويتك. قد يتم نشر نتائج هذا البحث.

هل يمكن الغاء مشاركتي دون موافقتي؟

بامكان الشخص المسوؤل عن البحث انهاء مشاركتك بالبحث دون موافقتك اذا كان الاستبيان الذي قدمتة غير مكتمل.

مع من يمكنني التحدث؟

إذا كان لديك اسئله أو مخاوف أو شكاوى ، أو تعتقد أن البحث قد أضر بك، يجب عليك التحدث إلى فريق البحث:

الباحث الاساسي:

الاسم: سامينا سالم

القسم : الصيدلة وعلم الأدوية

رقم الهاتف: 713-743-1776

البريد الالكتروني: *ssalim2@central.uh.edu*

لقد قام مجلس المراجعة المؤسسية لجامعه هيوستن (IRB) بمراجعة هذا البحث والموافقة عليه. يمكنك أيضا التحدث معهم على الرقم (713-743-9204) , أوالبريد الالكتروني *cphs@central.uh.edu* إذا:

- **لم يتم الرد على أسئلتك أو مخاوفك أو شكواك من قبل فريق البحث.**
- **لم تتمكن من الوصول إلى فريق البحث.**
- **أردت التحدث إلى شخص أخربالاضافة لفريق البحث.**
- **كان لديك اسئله حول حقوقك كموضوع بحث.**
- **كنت ترغب في الحصول على معلومات أو تقديم مدخلات حول هذا البحث.**

لقد قرأت معلومات الموافقة وأوافق على المشاركة في البحث

* must provide value

○ Yes
○ No

Online Designer

Since this project is currently in PRODUCTION, changes will not be made in real time. Tell me more

Submit Changes for Review

Fields to be added: **0** / Total resulting field count: **129**
Fields to be deleted: **0** / Existing field count: **129**

Remove all drafted changes

View detailed summary of all drafted changes

Create snapshot of instruments

Last snapshot: never ?

VIDEO: How to use this page

This page allows you to build and customize your data collection instruments one field at a time. You may add new fields or edit existing ones. New fields may be added by clicking the Add Field buttons. You can begin editing an existing field by clicking on the **Edit** icon. If you decide that you do not want to keep a field, you can simply delete it by clicking on the **Delete** icon. To reorder the fields, simply **drag and drop** a field to a different position within the form below.

Return to list of instruments | Survey settings | « Previous instrument | Next instrument »

Current instrument: **Sociodemographics**

Preview instrument

Add Field | Add Matrix of Fields | Import from Field Bank

Variable: socio_1_1

Are you an Arab American who lives in the United States and is 18 years old or older?
NOTE: An Arab American is someone who identifies as ARAB and LIVES in the United States (you do not have to be a citizen)

هل أنت عربي أمريكي مقيم في الولايات المتحدة وعمرك 18 عاماً فما فوق؟

ملاحظة: الأمريكي العربي هو شخص يعرّف بنفسه بأنه عربي ويعيش في الولايات المتحدة الأمريكية (لا يشترط أن تكون مواطنأمريكي)

* must provide value

- ◯ Yes
- ◯ No

Add Field | Add Matrix of Fields | Import from Field Bank

Variable: socio_1

How old are you?

كم عمرك؟

* must provide value

Add Field | Add Matrix of Fields | Import from Field Bank

Variable: socio_2

What is your gender?

ما هو جنسك؟

* must provide value

- ◯ Male ذكر
- ◯ Female انثى
- ◯ Transgender man رجل عابر جنسيا
- ◯ Transgender woman إمرأة عابرة جنسياً
- ◯ Non-Binary غير ثنائي
- ◯ Other

Add Field | Add Matrix of Fields | Import from Field Bank

Variable: socio_2_1 *Branching logic:* [socio_2]="5"

Other specify
غير ذلك

Add Field | Add Matrix of Fields | Import from Field Bank

Variable: socio_3

How many years of education have you completed?

ما هو أعلى مستوى تعليمي حصلت عليه؟

* must provide value

- High school or less الثانوية العامة أو أقل
- Some college or associate's degree درجة الكلية أو الزمالة
- College degree شهادة جامعية
- Graduate degree شهادة الدراسات العليا

Add Field | Add Matrix of Fields | Import from Field Bank

Variable: socio_6

What is your zipcode?
NOTE: We are asking for your zipcode because we generally want to see how the health of Arab Houstonians is different in different regions of the Houston. This can help us understand how to improve health services and outreach. Your responses will remain anonymous. We will not be able to identify you in any way.

ما هو الرمز البريدي الخاص بك؟
ملاحظة: نطلب الرمز البريدي الخاص بك ألننا نريد أن نكتشف بشكل عام كيف قد تختلف صحة العرب األمريكيين في المناطق المختلفة .من الواليات المتحدة. يمكن أن يساعدنا ذلك على فهم كيفية وامكانية تحسين الخدمات الصحية والتوعية. ستبقى ردودك مجهولة الهوية .لن نتمكن من التعرف عليك بأي شكل من األشكال

* must provide value

Add Field | Add Matrix of Fields | Import from Field Bank

Variable: socio_7

What is your marital status?

ما هي حالتك الاجتماعية؟

* must provide value

- Married متزوج
- Widowed أرمل
- Divorced مطلق
- Separated منفصل
- Never married غير متزوج
- Living with a partner أعيش مع شريكي
- Other غير ذلك

Add Field | Add Matrix of Fields | Import from Field Bank

Variable: socio_9

Which of the following best describes the place that you live?

أي مما يلي يصف المكان الذي تعيش فيه بالشكل الأفضل؟

* must provide value

- I or someone in my household owns the place where I live أنا أو شخص ما في بيتي يمتلك المكان الذي أعيش فيه
- I rent the place where I live أنا أستأجر المكان الذي أعيش فيه
- I occupy the place where I live without payment or cash rent أنا أسكن في المكان الذي أعيش فيه بدون دفع أو إيجار نقدي

Add Field | Add Matrix of Fields | Import from Field Bank

Variable: socio_10

We will now ask you about the total household income for all persons who live in your home, including income from sources such as wages, salaries, Social Security or retirement benefits, help from relatives and so fourth.

What is your annual household income before taxes?

سوف نسألك الآن عن إجمالي دخل الأسرة لجميع الأشخاص الذين يعيشون في منزلك، بما في ذلك مصادر الدخل مثل الأجور والرواتب، والضمان الاجتماعي أو استحقاقات التقاعد، والمساعدة من الأقارب وما إلى ذلك.

ما هو دخل أسرتك السنوي قبل الضرائب

- $0 to less than $5,000
- $5,000 to less than $15,000
- $15,000 to less than $25,000
- $25,000 to less than $35,000
- $35,000 to less than $45,000
- $45,000 to less than $55,000
- $55,000 to less than $65,000
- $65,000 to less than $75,000
- $75,000 to less than $100,000
- $100,000 and over 100 (>$100,000)

* must provide value

Add Field | Add Matrix of Fields | Import from Field Bank

Variable: socio_11

Are you a United States citizen?

هل أنت مواطن أمريكي؟

* must provide value

- Yes نعم
- No لا
- I don't know لا أعلم

Add Field | Add Matrix of Fields | Import from Field Bank

Variable: socio_12

Were you born in the United States?

هل ولدت في الولايات المتحدة؟

* must provide value

- Yes
- No

Add Field | Add Matrix of Fields | Import from Field Bank

Variable: socio_13 *Branching logic:* [socio_12]="2"

Where were you born?

أين ولدت؟

* must provide value

Add Field | Add Matrix of Fields | Import from Field Bank

Variable: socio_14 *Branching logic:* [socio_12]="2"

How long have you lived in the United States?

كم مدة إقامتك في الولايات المتحدة؟

* must provide value

- 0-2 years (سنوات)
- 3-5 years (سنوات)
- 6-10 years (سنوات)
- 10-14 years (سنوات)
- 15-20 years (سنوات)
- 20+ years (> 10 سنوات)

Add Field | Add Matrix of Fields | Import from Field Bank

Variable: socio_15

Were your parents born in the United States?

هل والداك من مواليد الولايات المتحدة؟

* must provide value

- Yes
- No

Add Field | Add Matrix of Fields | Import from Field Bank

Variable: socio_15_1 *Branching logic:* [socio_15]="0"

Where was your mother born?

أين ولدت والدتك؟

* must provide value

Add Field | Add Matrix of Fields | Import from Field Bank

Variable: socio_15_2 *Branching logic:* [socio_15]="0"

Where was your father born?

أين ولد والدك؟

* must provide value

Add Field | Add Matrix of Fields | Import from Field Bank

Variable: socio_17

What is your race? (select all that apply)

(ما هو عرقك؟ (اختر كل ما ينطبق)

* must provide value

- ☐ White أبيض
- ☐ Middle Eastern or North African الشرق الأوسط أو شمال أفريقيا
- ☐ Black or African American أسود أو أفريقي أمريكي
- ☐ American Indian or Alaska Native أمريكي هندي أو من سكان الاسكا
- ☐ Asian آسيوي
- ☐ Native Hawaiian or Pacific Islander من سكان هاواي أو جزر المحيط الهادئ
- ☐ Other غير ذلك

Add Field | Add Matrix of Fields | Import from Field Bank

Variable: socio_17_1 *Branching logic:* [socio_17(6)]="1"

Other specify
غير ذلك

Add Field | Add Matrix of Fields | Import from Field Bank

Variable: socio_18

How well do you understand English?

ما هو مدى فهمك للغة الإنجليزية؟

* must provide value

- ◯ Extremely well ممتاز
- ◯ Very well جيد جداً
- ◯ Moderately well جيد باعتدال
- ◯ Slightly well جيد قليلاً
- ◯ Not well at all ليس جيداً أبداً

Add Field | Add Matrix of Fields | Import from Field Bank

Variable: socio_19

How well do you understand Arabic?

ما هو مدى فهمك للغة العربية؟

* must provide value

- ◯ Extremely well ممتاز
- ◯ Very well جيد جداً
- ◯ Moderately well جيد باعتدال
- ◯ Slightly well جيد قليلاً
- ◯ Not well at all ليس جيداً أبداً

Add Field | Add Matrix of Fields | Import from Field Bank

Variable: socio_20

What is your religion?

ما هي ديانتك؟

* must provide value

- ◯ Christian مسيحي
- ◯ Muslim مسلم
- ◯ Jewish يهودي
- ◯ Hindhu هندوسي
- ◯ Buddhist بوذي
- ◯ Atheist ملحد
- ◯ Other غير ذلك

Add Field | Add Matrix of Fields | Import from Field Bank

Variable: socio_20_1 *Branching logic:* [socio_20]="6"

Other specify
حدد, غير ذلك

* must provide value

Add Field | Add Matrix of Fields | Import from Field Bank

Variable: socio_21

How important is your religion to you?

ما هي أهمية ديانتك بالنسبة إليك؟

* must provide value

- ◯ Extremely important مهمة للغاية
- ◯ Very important مهمة جداً
- ◯ Moderately important مهمة بشكل معتدل
- ◯ Slightly important مهمة قليلاً
- ◯ Not at all important ليست مهمة أبداً

Add Field | Add Matrix of Fields | Import from Field Bank

Variable: socio_22

Which of these options best describes your current work situation?

أي من هذه الخيارات يصف وضع عملك الحالي بشكل أفضل؟

* must provide value

- Working full-time أعمل بدوام كامل
- Working part-time أعمل بدوام جزئي
- Looking for work or unemployed باحث عن عمل أو عاطل عن العمل
- Retired متقاعد
- A homemaker رب منزل
- A student طالب
- On maternity or paternity leave في إجازة الأمومة أو الأبوة
- On illness or sick leave في حالة مرض أو في إجازة المرضية
- On disability في حالة الإعاقة
- Other غير ذلك

Add Field | Add Matrix of Fields | Import from Field Bank

Variable: socio_22_1 *Branching logic:* [socio_22]="10"

Other specify
حدد, غير ذلك

* must provide value

Add Field | Add Matrix of Fields | Import from Field Bank

Variable: socio_23

Are you covered by any kind of health insurance or some other kind of health care plan?

هل أنت مغطى بأي نوع من التأمين الصحي أو أي نوع آخر من خطط الرعاية الصحية؟

* must provide value

- Yes نعم
- No لا
- I don't know لا أعلم

Add Field | Add Matrix of Fields | Import from Field Bank

Variable: socio_23_1 *Branching logic:* [socio_23]="1" or [socio_23]="3"

What type of health insurance or health care coverage do you have?

ما هي أنواع التأمينات الصحية أو الرعاية الصحية التي تمتلكها؟

* must provide value

- Health insurance paid for by an employer or a union تأمين صحي يدفعه صاحب العمل أو النقابة
- Health insurance you or your family pays for yourself تأمين الصحي تدفعه أنت أو عائلتك لنفسك
- Medicare ميديكير
- Medicaid ميديكيد
- Some other kind of health insurance نوع آخر من التأمين الصحي
- No coverage of any type لا يوجد أي تأمين من أي نوع
- I don't know لا أعلم

Add Field | Add Matrix of Fields | Import from Field Bank

Online Designer

Since this project is currently in PRODUCTION, changes will not be made in real time. Tell me more

Submit Changes for Review

Fields to be added: **0** / Total resulting field count: **129**
Fields to be deleted: **0** / Existing field count: **129**

Remove all drafted changes

View detailed summary of all drafted changes

Create snapshot of instruments

Last snapshot: never ?

VIDEO: How to use this page

This page allows you to build and customize your data collection instruments one field at a time. You may add new fields or edit existing ones. New fields may be added by clicking the Add Field buttons. You can begin editing an existing field by clicking on the **Edit** icon. If you decide that you do not want to keep a field, you can simply delete it by clicking on the **Delete** icon. To reorder the fields, simply **drag and drop** a field to a different position within the form below.

Return to list of instruments | Survey settings | « Previous instrument | Next instrument »

Current instrument: **Health Questions**

Preview instrument

Add Field | Add Matrix of Fields | Import from Field Bank

Variable: health_1

Would you say your health in general is excellent, very good, good, fair, or poor?

هل يمكنك القول أن صحتك العامة ممتازة، جيدة جداً، جيدة، لا بأس بها، أو سيئة؟

* must provide value

Add Field | Add Matrix of Fields | Import from Field Bank

Matrix group: diseases

Have you EVER been told by a doctor or other health professional that you had:

هل سبق تم إخبارك إطلاقاً من قبل طبيب أو اختصاصي صحي آخر بأنه لديك

Variable	Item	Yes نعم	No لا	Don't know لا أعلم
health_2	**Hypertension (high blood pressure)** ارتفاع ضغط الدم * must provide value	○	○	○
health_3	**High cholesterol** ارتفاع الكوليسترول * must provide value	○	○	○
health_4	**Coronary heart disease** أمراض القلب التاجية * must provide value	○	○	○
health_5	**Heart attack** أزمة قلبية * must provide value	○	○	○
health_6	**Stroke** سكتة دماغية * must provide value	○	○	○
health_7	**Asthma** ربو * must provide value	○	○	○
health_8	**Cancer** سرطان * must provide value	○	○	○

Variable: health_9

Pre-diabetes or borderline diabetes مقدمات السكري
* must provide value

Variable: health_10

Diabetes السكري
* must provide value

Variable: health_11

Chronic obstructive pulmonary disease (COPD), emphysema, or chronic bronchitis مرض الانسداد الرئوي المزمن (COPD) ، وانتفاخ الرئة ، أو التهاب الشعب الهوائية المزمن
* must provide value

Variable: health_12

Arthritis, rheumatoid arthritis, gout, lupus, or fibromyalgia? التهاب المفاصل ، التهاب المفاصل الروماتزم ، النقرس ، الذئبة ، أو الألم العضلي الليفي
* must provide value

Variable: health_13

Dementia, including Alzheimer's disease الخرف ، بما في ذلك مرض الزهايمر
* must provide value

Variable: health_14

Obesity السمنة المفرطة
* must provide value

Variable: health_15

Other:
* must provide value

Add Field | Add Matrix of Fields | Import from Field Bank

Variable: health_8_1 *Branching logic:* [health_8]="1"

What kind of cancer?

ما هو نوع السرطان؟

* must provide value

- ☐ Bladder المثانة
- ☐ Blood الدم
- ☐ Bone العظام
- ☐ Brain الدماغ
- ☐ Breast الثدي
- ☐ Cervix/Cervical عنق الرحم
- ☐ Colon القولون
- ☐ Esophagus/Esophageal المريء
- ☐ Gallbladder المرارة
- ☐ Kidney الكلى
- ☐ Larynx-trachea الحنجرة-القصبة الهوائية
- ☐ Leukemia لويكيميا
- ☐ Liver الكبد
- ☐ Lung الرئتين
- ☐ Lymphoma الغذد الليمفاوية
- ☐ Melanoma الجلد
- ☐ Mouth/tongue/lip الفم/اللسان/الشفاه
- ☐ Ovary/Ovarian المبيض
- ☐ Pancreas/Pancreatic البنكرياس
- ☐ Prostate البروستاتا
- ☐ Rectum/Rectal المستقيم
- ☐ Skin (non-melanoma) الجلد (غير الورم الميلاني)
- ☐ Skin (don't know what kind) الجلد (لا أعلم ما هو النوع)
- ☐ Soft tissue (muscle or fat) الأنسجة الرخوة (العضلات أو الدهون)
- ☐ Stomach المعدة
- ☐ Testis/Testicular الخصية
- ☐ Throat - pharynx الحلق - البلعوم
- ☐ Thyroid غدة درقية
- ☐ Uterus/Uterine الرحم
- ☐ Other غير ذلك
- ☐ Don't know لا أعلم

Add Field | Add Matrix of Fields | Import from Field Bank

Variable: health_10_1 *Branching logic:* [health_10]="1"

According to your doctor or other health professional, what type of diabetes do you have?

وفقًا لطبيبك أو أخصائي صحي آخر ، ما نوع مرض السكري الذي تعاني منه؟

* must provide value

- ○ Type 1 النوع الأول
- ○ Type 2 النوع الثاني
- ○ Other type of diabetes نوع آخر من السكري
- ○ I don't know لا أعلم

Add Field | Add Matrix of Fields | Import from Field Bank

Variable: health_16

Have you EVER been told by a doctor or other health professional that you had any type of anxiety disorder?

Different types of anxiety disorders include generalized anxiety disorder, social anxiety disorder, panic disorder, post-traumatic stress disorder, obsessive-compulsive disorder, or phobias.

هل سبق تم إخبارك من قبل طبيب أو اختصاصي صحي بأنه لديك أي نوع من اضطرابات القلق؟

تشمل الأنواع المختلفة من اضطرابات القلق اضطراب القلق العام ، واضطراب القلق الاجتماعي ، واضطراب الهلع ، واضطراب ما بعد الصدمة ، والوسواس القهري ، أو الرهاب.

* must provide value

- ○ Yes نعم
- ○ No لا
- ○ I don't know لا أعلم

Add Field | Add Matrix of Fields | Import from Field Bank

Variable: health_17

Have you EVER been told by a doctor or other health professional that you had any type of depression?

Different types of depression include major depressive disorder, bipolar depression, dysthymia, post-partum depression, and seasonal affective disorder.

هل أخبرك طبيب أو أخصائي صحي آخر على الإطلاق أنك مصاب بأي نوع من الاكتئاب؟

تشمل الأنواع المختلفة من الاكتئاب اضطراب الاكتئاب الشديد، والاكتئاب ثنائي القطب، واضطراب عسر المزاج ، واكتئاب ما بعد الولادة ، والاضطراب العاطفي الفصلي.

* must provide value

- ◯ Yes نعم
- ◯ No لا
- ◯ I don't know لا أعلم

Add Field | Add Matrix of Fields | Import from Field Bank

Matrix group: depression

In the past 2 weeks, how often have you been bothered by any of the following problems?

خلال الأسبوعين الماضيين، كم مرة انزعجت من أي من المشاكل التالية؟

	Not at all لم تحدث في أي يوم	Several days أيام عديدة	More than half the days أكثر من نصف الأيام	Nearly every day يومياً تقريباً
Variable: health_18 — **Little interest or pleasure in doing things** قلة الاهتمام أو المتعة في فعل الأشياء * must provide value	◯	◯	◯	◯
Variable: health_19 — **Feeling down, depressed, or hopeless** الشعور بالإحباط أو الاكتئاب أو اليأس * must provide value	◯	◯	◯	◯
Variable: health_20 — **Trouble falling or staying asleep, OR sleeping too much** صعوبة في النوم أو البقاء نائمًا ، أو النوم كثيرًا * must provide value	◯	◯	◯	◯
Variable: health_21 — **Feeling tired or having little energy** الشعور بالتعب أو قلة الطاقة * must provide value	◯	◯	◯	◯
Variable: health_22 — **Poor appetite OR overeating** ضعف الشهية أو الإفراط في تناول الطعام * must provide value	◯	◯	◯	◯
Variable: health_23 — **Feeling bad about yourself - or that you are a failure or have let yourself or your family down** الشعور بالسوء حيال نفسك - أو أنك فشلت أو خذلت نفسك أو عائلتك * must provide value	◯	◯	◯	◯
Variable: health_24 — **Trouble concentrating on things, such as reading the newspaper or watching television** صعوبة في التركيز على الأشياء ، مثل قراءة الصحف أو مشاهدة التلفزيون * must provide value	◯	◯	◯	◯

Variable: health_25

Moving or speaking so slowly that other people have noticed? Or the opposite - being so fidgety or restless that you have been moving around a lot more than usual هل تتحرك أو تتحدث ببطء شديد حتى أن الآخرين لاحظوا ذلك؟ أو العكس - أن تكون متململًا أو مضطربًا لدرجة أنك تتحرك كثيرًا أكثر من المعتاد
* must provide value

○ ○ ○ ○

Variable: health_26

Thoughts that you would be better off dead or of hurting yourself in some way أفكار أنك ستكون أفضل حالًا بعد الموت أو أن تؤذي نفسك بطريقة ما
* must provide value

○ ○ ○ ○

Add Field | Add Matrix of Fields | Import from Field Bank

Matrix group: anxiety

Over the past 2 weeks, how often have you been bothered by any of the following problems?

خلال الأسبوعين الماضيين، كم مرة انزعجت من أي من المشاكل التالية؟

Variable: health_27

	Not at all لم تحدث في أي يوم	Several days أيام عديدة	More than half the days أكثر من نصف الأيام	Nearly every day يومياً تقريباً
**Feeling nervous, anxious, or on edge** الشعور بالتوتر أو القلق أو على الحافة * must provide value	○	○	○	○
Variable: health_28 **Not being able to stop or control worrying** عدم القدرة على التوقف أو السيطرة على القلق * must provide value	○	○	○	○
Variable: health_29 **Worrying too much about different things** القلق الشديد بشأن أشياء مختلفة * must provide value	○	○	○	○
Variable: health_30 **Trouble relaxing** صعوبة الاسترخاء * must provide value	○	○	○	○
Variable: health_31 **Being so restless that it is hard to sit still** أن تكون مضطربًا للغاية بحيث يصعب عليك الجلوس * must provide value	○	○	○	○
Variable: health_32 **Becoming easily annoyed or irritable** الانزعاج بسهولة أو سرعة الانفعال * must provide value	○	○	○	○
Variable: health_33 **Feeling afraid as if something awful might happen** الشعور بالخوف كما لو أنك تتوقع حدوث شيء فظيع * must provide value	○	○	○	○

Add Field | Add Matrix of Fields | Import from Field Bank

Matrix group: covid

Has the coronavirus or COVID-19 outbreak been so frightening, horrible, or upsetting...
هل كان تفشي فيروس كورونا أو كوفيد-19 مخيفًا أو فظيعًا أو مزعجًا لدرجة

Variable: health_34

	Yes نعم	No لا
**that you had nightmares about it or thought about it when you did not want to? أنه كان لديك كوابيس عنه أو فكرت فيه عندما كنت لا تريد ذلك؟** * must provide value	○	○

Variable: health_35

**that you tried hard not to think about it, or went out of your way to avoid situations that reminded you of it? أنك حاولت جاهدًا ألا تفكر في الأمر، وحاولت تجنب المواقف التي ذكّرتك به؟** * must provide value	○	○

Variable: health_36

**that you were constantly on guard, watchful, or easily startled? أنك كنت في حالة التأهب، أو متيقظًا باستمرار ، أو منزعجًا بسهولة؟** * must provide value	○	○

Variable: health_37

**that you felt numb or detached from others, activities, or your surroundings? أنك شعرت بالخدر أو الانفصال عن الآخرين أو الأنشطة أو محيطك؟** * must provide value	○	○

Add Field | Add Matrix of Fields | Import from Field Bank

Variable: health_38

Because of a physical, mental, or emotional condition, do you have difficulty doing errands alone such as visiting a doctor's office or shopping?

بسبب حالة جسدية أو نفسية أو عاطفية، هل تجد صعوبة في القيام بمهام وحدك مثل زيارة عيادة الطبيب أو التسوق؟

* must provide value

- ○ No difficulty لا توجد صعوبة
- ○ Some difficulty توجد القليل من الصعوبة
- ○ A lot of difficulty توجد الكثير من الصعوبة
- ○ Cannot do at all لا أستطيع القيام بذلك أبداً
- ○ I don't know لا أعلم

Add Field | Add Matrix of Fields | Import from Field Bank

Variable: health_39

Because of a physical, mental, or emotional condition, do you have difficulty participating in social activities such as visiting friends, attending clubs and meetings, or going to parties?

بسبب حالة جسدية أو نفسية أو عاطفية، هل تجد صعوبة في المشاركة في الأنشطة الاجتماعية، مثل زيارة الأصدقاء، أو حضور الأندية والاجتماعات، أو الذهاب إلى الحفلات؟

* must provide value

- ○ No difficulty لا توجد صعوبة
- ○ Some difficulty توجد القليل من الصعوبة
- ○ A lot of difficulty توجد الكثير من الصعوبة
- ○ Cannot do at all لا أستطيع القيام بذلك أبداً
- ○ I don't know لا أعلم

Add Field | Add Matrix of Fields | Import from Field Bank

Variable: health_40

Do you smoke (cigarettes, hookah, other tobacco products)?

هل تدخن (تدخين السجائر، الأرجيلة، وغيرها من منتجات التبغ) ؟

* must provide value

- ○ Yes
- ○ No

Add Field | Add Matrix of Fields | Import from Field Bank

Variable: health_40_1 *Branching logic:* [health_40]="1"

How often do you smoke (cigarettes, hookah, other tobacco products)?

كم مرة تقوم بالتدخين (تدخين السجائر، الأرجيلة، وغيرها من منتجات التبغ) ؟

* must provide value

- ○ Every day كل يوم
- ○ Some days بعض الأيام
- ○ Not at all لا أدخن إطلاقاً

Add Field | Add Matrix of Fields | Import from Field Bank

Variable: health_41 *Branching logic:* [health_40]="1"

Since coronavirus or COVID-19 started, are you smoking more, the same, or fewer than you did before coronavirus started?

منذ بدء انتشار فيروس كورونا أو كوفيد-19، هل أصبحت تدخن أكثر، أو بنفس الكمية، أو أقل مما كنت قبل بداية انتشار فيروس كورونا؟

* must provide value

- Smoking more أدخن أكثر
- Smoking less أدخن أقل
- Smoking the same amount أدخن بنفس الكمية
- I have never smoked لا أدخن إطلاقاً

Add Field | Add Matrix of Fields | Import from Field Bank

Variable: health_42

Do you drink alcohol?

هل تشرب الكحول؟

* must provide value

- Yes
- No

Add Field | Add Matrix of Fields | Import from Field Bank

Variable: health_42_1 *Branching logic:* [health_42]="1"

How often do you drink alcohol?

كم مرة تشرب الكحول؟

* must provide value

- Every day كل يوم
- Some days بعض الأيام
- Not at all لا أدخن إطلاقاً

Add Field | Add Matrix of Fields | Import from Field Bank

Variable: health_43 *Branching logic:* [health_42]="1"

Since coronavirus or COVID-19 started, are you drinking more, the same, or less alcohol than you did before coronavirus started?

منذ بدء انتشار فيروس كورونا أو كوفيد-19، هل أصبحت تشرب الكحول أكثر، أو بنفس الكمية، أو أقل مما كنت قبل بداية انتشار فيروس كورونا؟

* must provide value

- Drinking more alcohol أشرب الكحول أكثر
- Drinking less alcohol أشرب الكحول أقل
- Drinking the same amount of alcohol أشرب الكحول بنفس الكمية
- I have never had alcohol لا أشرب الكحول إطلاقاً

Add Field | Add Matrix of Fields | Import from Field Bank

Online Designer

Since this project is currently in PRODUCTION, changes will not be made in real time. Tell me more

Submit Changes for Review

Fields to be added: **0** / Total resulting field count: **129**
Fields to be deleted: **0** / Existing field count: **129**

Remove all drafted changes

View detailed summary of all drafted changes

Create snapshot of instruments

VIDEO: How to use this page

Last snapshot: never ?

This page allows you to build and customize your data collection instruments one field at a time. You may add new fields or edit existing ones. New fields may be added by clicking the Add Field buttons. You can begin editing an existing field by clicking on the **Edit** icon. If you decide that you do not want to keep a field, you can simply delete it by clicking on the **Delete** icon. To reorder the fields, simply **drag and drop** a field to a different position within the form below.

Return to list of instruments | Survey settings | « Previous instrument | Next instrument »

Current instrument: **COVID 19 Vaccination**

Preview instrument

Add Field | Add Matrix of Fields | Import from Field Bank

Variable: vaccine_3

Are you, or have you been, infected with the novel coronavirus (COVID-19)?

هل أنت الآن مصاب، أو كنت مصاباً , بفيروس كورونا المستجد (كوفيد-19)؟

* must provide value

- Yes, tested and the result was positive (نعم، تم فحصي ونتيجة الفحص إيجابية)
- Yes, suspected but not confirmed by a test (نعم، تم الاشتباه ولكن دون التأكد من خلال الفحص)
- No, tested and the result was negative (لا، تم الفحص ونتيجة الفحص سلبية)
- No (لا)
- Don't know (لا أعلم)
- Prefer not to say (أفضل عدم الإجابة)

Add Field | Add Matrix of Fields | Import from Field Bank

Variable: vaccine_4

Do you know people in your immediate social environment (family, friends, relatives etc.) who are or have been infected with the novel coronavirus (COVID-19)?

هل تعرف أشخاصاً في بيئتك الاجتماعية المباشرة (العائلة، الأصدقاء، الأقارب، الخ.) مصابون أو كانوا مصابين بفيروس كورونا المستجد (كوفيد-19)؟

* must provide value

- Yes, confirmed نعم، مؤكدة بفحص
- Yes, suspected but not confirmed by a test نعم، مشتبه بهم لكن دون التأكد من خلال الفحص
- No, tested and the result was negative لا، تم الفحص والنتيجة سلبية
- No لا
- Don't know لا أعلم
- Prefer not to say أفضل عدم الإجابة

Add Field | Add Matrix of Fields | Import from Field Bank

Variable: vaccine_5

How would you rate your knowledge level on how to prevent spread of the novel coronavirus?

كيف يمكنك تقييم درجة معرفتك بكيفية منع انتشار فيروس كورونا المستجد؟

* must provide value

- Excellent ممتازة
- Good جيدة
- Average متوسطة
- Poor ضعيفة
- Terrible سيئة

Add Field | Add Matrix of Fields | Import from Field Bank

Variable: vaccine_6

What do you consider your own probability/risk of getting infected with the novel coronavirus?

كم تقدر احتمالية/خطورة إصابتك بعدوى فيروس كورونا المستجد؟

* must provide value

- Extremely likely محتمل جداً
- Somewhat likely محتمل قليلاً
- Neither likely nor unlikely ليس محتمل وليس غير محتمل
- Somewhat unlikely غير محتمل قليلاً
- Extremely unlikely غير محتمل جداً
- I don't know لا أعلم

Add Field | Add Matrix of Fields | Import from Field Bank

Variable: vaccine_7

How severe would contracting the novel coronavirus be for you? (How seriously ill do you think you would be?)

ما هي مدى خطورة إصابتك بعدوى فيروس كورونا المستجد بالنسبة إليك؟ (كيف تتوقع أن تكون إصابتك خطيرة)؟

* must provide value

- ◯ Extremely severe خطيرة للغاية
- ◯ Somewhat severe خطيرة نوعاً ما
- ◯ Neither severe nor mild ليست خطيرة أو خفيفة
- ◯ Somewhat mild خفيفة نوعاً ما
- ◯ Extremely mild خفيفة للغاية
- ◯ I don't know لا أعلم

Add Field | Add Matrix of Fields | Import from Field Bank

Variable: vaccine_8

I know how to protect myself from coronavirus.

أعرف كيفية حماية نفسي من فيروس كورونا.

* must provide value

- ◯ Strongly agree موافق بشدة
- ◯ Somewhat agree موافق نوعاً ما
- ◯ Neither agree nor disagree لست موافق أو غير موافق
- ◯ Somewhat disagree غير موافق نوعاً ما
- ◯ Strongly disagree غير موافق بشدة

Add Field | Add Matrix of Fields | Import from Field Bank

Variable: vaccine_9

For me avoiding an infection with the novel coronavirus in the current situation is...

أعتبر تجنب الإصابة بعدوى فيروس كورونا المستجد في الوضع الحالي أمر...

* must provide value

- ◯ Extremely easy سهل للغاية
- ◯ Somewhat easy سهل نوعاً ما
- ◯ Neither easy nor difficult ليس سهلاً أو صعباً
- ◯ Somewhat difficult صعب نوعاً ما
- ◯ Extremely difficult صعب للغاية
- ◯ I don't know لا أعلم

Add Field | Add Matrix of Fields | Import from Field Bank

Matrix group: confidence

How much confidence do you have in the below individuals and organizations that they can handle the novel coronavirus well?

ما هي نسبة ثقتك بالأشخاص والمنظمات أدناه حول قدرتهم على التعامل مع فيروس كورونا المستجد بشكل جيد؟

	A great deal نسبة عالية جدا	A lot نسبة كبيرة	A moderate amount نسبة متوسطة	A little نسبة قليلة	None at all لا ثقة إطلاقا
Variable: vaccine_10<br>**Your family doctor طبيب العائلة**<br>* must provide value	◯	◯	◯	◯	◯
Variable: vaccine_11<br>**Your employer رئيس عملك**<br>* must provide value	◯	◯	◯	◯	◯
Variable: vaccine_12<br>**Media الإعلام**<br>* must provide value	◯	◯	◯	◯	◯
Variable: vaccine_13<br>**Hospitals المستشفيات**<br>* must provide value	◯	◯	◯	◯	◯
Variable: vaccine_14<br>**The president الرئيس**<br>* must provide value	◯	◯	◯	◯	◯
Variable: vaccine_15<br>**Local public health authorities سلطات الصحة العامة**<br>* must provide value	◯	◯	◯	◯	◯
Variable: vaccine_16<br>**Schools المدارس**<br>* must provide value	◯	◯	◯	◯	◯

Variable: vaccine_17

**Universities الجامعات** * must provide value	○	○	○	○	○

Variable: vaccine_18

**Government الحكومة** * must provide value	○	○	○	○	○

Variable: vaccine_19

**Police الشرطة** * must provide value	○	○	○	○	○

Variable: vaccine_20

**Church or mosque الكنيسة أو المسجد** * must provide value	○	○	○	○	○

Add Field | Add Matrix of Fields | Import from Field Bank

Variable: vaccine_21

I follow the recommendations from local and national authorities to prevent spread of novel coronavirus.

.أقوم باتباع توصيات الجهات المحلية والدولية للحد من انتشار فيروس كورونا المستجد

* must provide value

- ○ Very much so أقوم بذلك كثيراً
- ○ A moderate amount أقوم بذلك بشكل معتدل
- ○ A little أقوم بذلك قليلاً
- ○ Not at all لا أقوم بذلك إطلاقاً

Add Field | Add Matrix of Fields | Import from Field Bank

Variable: vaccine_21_1

Did you receive COVID-19 vaccine?
هل تلقيت مطعوم فيروس كوفيد-19

* must provide value

- ○ Yes
- ○ No

Add Field | Add Matrix of Fields | Import from Field Bank

Variable: vaccine_21_1_1 *Branching logic:* [vaccine_21_1]="1"

Which vaccine did you receive?
أي مطعوم تلقيت؟

- ○ Moderna
- ○ Pfizer
- ○ Johnson and Johnson
- ○ Other
- ○ I don't know

Add Field | Add Matrix of Fields | Import from Field Bank

Matrix group: opinion

Please give your opinion on the following statements:

نرجو تقديم رأيك حول الجمل التالية

Variable: vaccine_23

	Strongly agree موافق بشدة	Somewhat agree موافق نوعاً ما	Neither agree nor disagree لست موافقاً أو غير موافق	Somewhat disagree غير موافق نوعاً ما	Strongly disagree غير موافق بشدة
**From now on, anyone moving in a public area should be required to wear a face mask.** منذ هذه اللحظة، يجب على أي شخص يتنقل في المناطق العامة أن يرتدي كمامة الوجه * must provide value	○	○	○	○	○
Variable: vaccine_24					
**More tests for coronavirus infection should be carried out in the population** يجب إجراء المزيد من الفحوصات لفيروس كورونا للسكان * must provide value	○	○	○	○	○
Variable: vaccine_25					
**I think that restrictions currently being implemented are greatly exaggerated.** أعتقد أن القيود المفروضة حالياً مبالغ فيها كثير * must provide value	○	○	○	○	○

Variable: vaccine_26

You should only be allowed to leave your house for professional, health, or urgent reasons. يجب أن يسمح بمغادرة المنزل لاسباب مهنية، صحية، أو عاجلة
* must provide value

Add Field | Add Matrix of Fields | Import from Field Bank

Matrix group: done_during_pandemic

Have you done the following during the pandemic?

هل قمت بما يلي خلال انتشار الوباء...؟

Variable	Item	I already did that فعلت ذلك مسبقاً	I plan to do that أخطط لفعل ذلك	I don't plan to do that لا أخطط لفعل ذلك
vaccine_27	**Bought food supplies on a large scale** شراء المؤن الغذائية بكميات كبيرة * must provide value	○	○	○
vaccine_28	**Exercised less than I usually do** ممارسة الرياضة أقل من العادة * must provide value	○	○	○
vaccine_29	**Ate more unhealthy food than I usually do** تناول الأطعمة غير الصحية أكثر من العادة * must provide value	○	○	○
vaccine_30	**Avoided going to the doctor with issues that could be postponed (e.g. vaccination or a check-up)** تجنب الذهاب إلى الطبيب بسبب مشاكل يمكن تأجيلها (مثل التطعيم أو الفحص) * must provide value	○	○	○
vaccine_31	**Asked family members or friends not to visit me** الطلب من أفراد العائلة أو الأصدقاء عدم زيارتي * must provide value	○	○	○
vaccine_32	**Bought personal protective equipment (masks, gloves)** شراء معدات الوقاية الشخصية (الأقنعة والقفازات) * must provide value	○	○	○

Add Field | Add Matrix of Fields | Import from Field Bank

Online Designer

Since this project is currently in PRODUCTION, changes will not be made in real time. Tell me more

Submit Changes for Review

Fields to be added: **0** / Total resulting field count: **129**
Fields to be deleted: **0** / Existing field count: **129**

Remove all drafted changes

View detailed summary of all drafted changes

Create snapshot of instruments

Last snapshot: never ?

VIDEO: How to use this page

This page allows you to build and customize your data collection instruments one field at a time. You may add new fields or edit existing ones. New fields may be added by clicking the Add Field buttons. You can begin editing an existing field by clicking on the **Edit** icon. If you decide that you do not want to keep a field, you can simply delete it by clicking on the **Delete** icon. To reorder the fields, simply **drag and drop** a field to a different position within the form below.

Return to list of instruments | Survey settings | « Previous instrument

Current instrument: **COVID-19 Vaccination 2**

Preview instrument

Add Field | Add Matrix of Fields | Import from Field Bank

Variable: covid2_1

How susceptible do you consider yourself to an infection with the novel coronavirus?

كم تقدر قابلية إصابتك بعدوى فيروس كورونا المستجد؟

* must provide value

- Extremely susceptible قابل جداً
- Somewhat susceptible قابل قليلاً
- Neither susceptible or not ليس قابل وليس غير قابل
- Somewhat not susceptible غير قابل قليلاً
- Extremely unsusceptible غير قابل جداً

Add Field | Add Matrix of Fields | Import from Field Bank

Variable: covid2_2

Not seeing my family living outside my own home is emotionally:

الامتناع عن رؤية أفراد عائلتي خارج منزلي الخاص هو أمر:

* must provide value

- Extremely easy سهل للغاية
- Somewhat easy سهل نوعاً ما
- Neither easy nor difficult ليس سهلاً أو صعباً
- Somewhat difficult صعب نوعاً ما
- Extremely difficult صعب للغاية

Add Field | Add Matrix of Fields | Import from Field Bank

Variable: covid2_3

Not seeing my friends is emotionally:

الامتناع عن رؤية أصدقائي هو أمر:

* must provide value

- Extremely easy سهل للغاية
- Somewhat easy سهل نوعاً ما
- Neither easy nor difficult ليس سهلاً أو صعباً
- Somewhat difficult صعب نوعاً ما
- Extremely difficult صعب للغاية

Add Field | Add Matrix of Fields | Import from Field Bank

Variable: covid2_4

The novel coronavirus to me feels:

أشعر بأن فيروس كورونا المستجد:

* must provide value

- Close to me قريب مني
- Neither close nor far ليس قريباً ولا بعيداً
- Far from me بعيد عني

Add Field | Add Matrix of Fields | Import from Field Bank

Variable: covid2_5

The novel coronavirus to me feels like it is

أشعر بأن انتشار فيروس كورونا بالنسبة لي:

* must provide value

- Spreading slowly ينتشر ببطء
- Spreading neither slowly or fast لا ينتشر بسرعة ولا ينتشر ببطء
- Spreading fast ينتشر بسرعة

Add Field | Add Matrix of Fields | Import from Field Bank

Variable: covid2_6

The novel coronavirus is:

إن فيروس كورونا المستجد هو:

* must provide value

- Something I think about all the time أمر أفكر به في جميع الأوقات
- Something I think about sometimes أمر أفكر به في بعض الأحيان
- Something I almost never think about أمر أكاد لا أفكر به إطلاقاً

Add Field | Add Matrix of Fields | Import from Field Bank

Variable: covid2_7

The novel coronavirus is:

إن فيروس كورونا المستجد هو:

* must provide value

- Media hyped أحدث ضجة إعلامية
- Not media hyped لم يحدث ضجة إعلامية
- Not media hyped enough لم يحدث ضجة إعلامية بالشكل الكافي

Add Field | Add Matrix of Fields | Import from Field Bank

Variable: covid2_8

The novel coronavirus is:

إن فيروس كورونا المستجد هو:

* must provide value

- Worrying مثير للقلق
- Not worrying غير مثير للقلق
- Neither لا شيء مما ذكر

Add Field | Add Matrix of Fields | Import from Field Bank

Variable: covid2_9

The novel coronavirus is:

إن فيروس كورونا المستجد هو:

* must provide value

- Stressful مثير للتوتر
- Not stressful غير مثير للتوتر
- Neither لا شيء مما ذكر

Add Field | Add Matrix of Fields | Import from Field Bank

Variable: covid2_10

The novel coronavirus is:

إن فيروس كورونا المستجد هو:

* must provide value

- Something that is making me depressed (أمر يجعلني أشعر بالاكتئاب)
- Something that is not affecting my mood (أمر لا يؤثر بحالتي المزاجية)
- Neither (لا شيء مما ذكر)

Add Field | Add Matrix of Fields | Import from Field Bank

Variable: covid2_11

Thank you for taking the time to complete the Survey for Arab Health in Houston. Remember that your responses will remain anonymous and secure. If you have any questions or concerns about your participation please contact the principal investigator Dr. Samina Salim (ssalim@uh.edu).

Feel free to send the survey to others!

نشكرك على وقتك لتعبئة استبيان صحة العرب في الولايات المتحدة. . تذكر أن إجاباتك ستبقى مجهولة الهوية ومحفوظة بأمان. إذا كان لديك أي أسئلة حول مشاركتك، نرجو التواصل مع الباحثة الرئيسية الدكتورة سامينا سالم (ssalim@uh.edu).

Add Field | Add Matrix of Fields | Import from Field Bank
